# Digital Stress-Preventive Management Competencies: Definition, Identification and Tool Development for Research and Practice

**DOI:** 10.3390/ijerph22020267

**Published:** 2025-02-12

**Authors:** Glauco Cioffi, Cristian Balducci, Stefano Toderi

**Affiliations:** 1 Department of Psychology, University of Bologna, 40127 Bologna, Italy; stefano.toderi@unibo.it; 2 Department for Life Quality Studies, University of Bologna, 47921 Rimini, Italy; cristian.balducci3@unibo.it

**Keywords:** digital leadership, remote work, psychosocial risks, well-being, self-other agreement

## Abstract

The digital transformation of work and the rise of remote workers (RWs) are gaining growing interest in occupational health science. However, research on managers’ role in well-being can be developed more. Aiming to bridge this gap, this study first defines and explores the Digital Stress-Preventive Management Competencies (DMCs) and then develops and validates an indicator tool with a three-phase procedure. Phase 1 consisted of a literature review and interviews with experts to identify DMCs, followed by item generation, content analysis and competencies conceptualization. Phase 2 was devoted to tool validation, comprising exploratory and confirmatory factor analysis with 247 RWs. Phase 3 explored the concurrent validity by investigating the relationship between DMCs and psychosocial factors via structural equation modeling (sample Phase 2) and polynomial regression with response surface analysis on 50 manager–team dyads (RWs 218). Two key competencies were identified: supportive ICT-mediated interaction (SIMI) and avoidance of abusive ICT adoption (AAIA). The final nine-item tool revealed a two-factor structure and good psychometric properties. SIMI was associated with superior support and role, while AAIA was linked to demands and control reported by RWs. These findings suggest that the DMCs identified and the related tool have potential applications in future organizational intervention content and for research purposes.

## 1. Introduction

The digital transformation of work is reshaping managerial roles, making digital leadership a critical factor for employee well-being and organizational sustainability [1,2,3,4]. As remote and hybrid work arrangements become the norm [5,6], managers must navigate new challenges, including ICT-mediated communication, trust-building, and work–life balance management [7,8].

Recent bibliographic reviews highlighted the growing interest among scholars in examining the requisite skills for supervisors in digital contexts [9,10]. Nevertheless, existing research has predominantly focused on supervisors’ digital competencies and their ability to promote team performance (e.g., [11,12,13,14,15,16,17,18,19,20]). Like the “traditional” (i.e., face-to-face) leadership literature, these studies acknowledge the performance-oriented approaches; however, they tend to overlook specific behaviors crucial for the health and well-being of employees [21,22,23]. Therefore, in the present study, we aim to advance our understanding of supervisors’ competencies in the context of digital work by adopting a health and well-being perspective. Specifically, this study aims to explore the Digital Stress-Preventive Management Competencies (DMCs)—a set of supervisory behaviors that foster a positive psychosocial work environment in remote teams—and develop and validate a tool to measure the latent construct. Such effort may contribute to the existing literature on hybrid or full-remote team management for workplace health, and it could be helpful for future practical (i.e., organizational intervention) and theoretical (i.e., research) applications.

The original value of our contribution lies in the fact that other existing approaches for constructing an index of digital leadership focused on the educational field [16,17] were mainly focused on performance [14,15,18], were based on a survey of the supervisors themselves [24], or they did not focus on management competencies related to optimizing psychosocial factors [25]. Thus, this study contributes to both the occupational health psychology and digital leadership literature by presenting a validated measurement tool and offering practical insights for organizational interventions, supervisors’ training, and digital work policies. Rather than aiming to replace broader digital leadership models, it seeks to complement them, particularly when addressing psychosocial risk factors in digital work contexts.

To do so, we designed and implemented a three-phase procedure. Phase 1 involved a literature review and interviews with subject matter experts to identify DMCs. Phase 2 focused on tool validation. Lastly, Phase 3 explored the concurrent validity of the new tool. The latter was carried out by investigating the relationship between DMCs and psychosocial factors with only employees-level data, also with a multisource view including manager–team (dis)agreement. The study structure is as follows: First, the next sections include (1.1) Stress-Preventive Management Competencies and (1.2) Conceptualization of Digital, Virtual and e-leadership and the Digital Stress-Preventive Management Competencies. Then, the present study (1.3) and the methodology (2) will be described in detail. Lastly, we state that the words manager/supervisor/leader and competence/skill are used interchangeably in this manuscript.

### 1.1. Stress-Preventive Management Competencies

Work-related stress and psychosocial risks pose significant challenges that require continuous monitoring and prevention due to their wide-ranging impact on individuals, organizations, and the economy [26,27,28,29]. Their effects manifest at multiple levels, including individual consequences such as sleep disorders, organizational issues like absenteeism, and broader societal impacts, such as fluctuations in Gross Domestic Product [30]. Accordingly, the ONU Agenda 2030 purposes ensuring health and promoting well-being while creating quality employment opportunities as two critical objectives to achieve global sustainable development, and many countries adopted legislative actions to control their propagation [31]. Given the complexity of the phenomenon, effective prevention and well-being enhancement require multi-stakeholder collaboration, structured and participatory approaches, and multilevel, preventive interventions (e.g., [27,28,32,33,34,35,36,37]). However, among all workplace actors, supervisors play a pivotal role in shaping the psychosocial work environment. Through their responsibilities lie in task design, work communication and organization, supervisors significantly influence the optimization—or mismanagement—of psychosocial factors. Furthermore, they are central to intervention strategies, organizational development, and change management processes [21,38,39]. As a result, initiatives aimed at fostering effective leadership behaviors have been recognized as both impactful [40,41] and highly recommended [28,42] in workplace stress-prevention frameworks.

To date, three approaches have explicitly considered employees’ health promotion and psychosocial risk prevention as leadership tasks. These frameworks investigated supervisors’ daily role-related management behaviors, specifically targeting their indirect impact on the health and well-being of employees (distal outcomes) via the improved quality of the psychosocial work environment (proximal outcome). The view underlying this field of study is that “stress management is a part of normal general management activities” for supervisors ([43], p. 11) and that “good supervision is more than a nice to have” ([44], p. 112). The approach that paved the way was proposed by Gilbreath and Benson [45]. A few years later, the Management Competencies for Preventing and Reducing Stress at Work (MCPARS) framework was developed and preliminarily tested by Yarker et al. [43,46,47]. More recently, St-Hilaire et al. suggested the Managerial Practices to Reduce Psychosocial Risk Exposure framework, which significantly overlaps with MCPARS [48]. However, the MCPARS is the framework that received more empirical attention.

This latter was developed by referencing the Management Standards (MS) approach to identify stress-preventive management behaviors [49,50]. Particularly, the MS claimed ‘states to be achieved’ as ideal work-related situations in six key psychosocial factors: demands (i.e., workload, work patterns); control (i.e., autonomy on the job, decision latitude); support (i.e., encouragement and resources provided by colleagues and supervisors) distinguishable into colleague’s support and supervisors’ support; relationships (i.e., the promotion of favorable working conditions to avoid conflict); role (i.e., role understanding, the avoidance of role conflict); and change (i.e., the management and communication of change). As a result, four key management competencies were identified: (1) Being Respectful and Responsible; (2) Managing and Communicating existing and future Work; (3) Reasoning/Managing Difficult Situations; and lastly, (4) Managing the Individual within the Team.

Notably, the four management competencies rated by employees were found to be linked to the psychosocial factors of MS—resilience, work engagement and workplace bullying perceptions [51,52,53,54]—while those self-assessed by the supervisors were linked to employees’ well-being through the mediating influence of the employees’ psychosocial work environment [55]. Additionally, two recent studies investigated the effect of manager–team (dis)agreement on the MCPARS competencies, highlighting the need to foster high-level agreement on the four competencies to prevent psychosocial risks and promote the job performance, mental health and well-being of team members [56,57]. These studies are particularly remarkable since self-ratings of leadership skills alone are not good predictors of a leader’s effectiveness [58,59,60], and researchers generally agree on leadership as being jointly established by leaders and followers [61,62,63]. Thus, multisource data involving multiple social actors’ perceptions and comparisons between ratings (i.e., Self-other agreement studies) are recommended to investigate leader effectiveness and outcomes better.

Importantly, the “traditional” (i.e., face-to-face) competencies do not disappear in digital workspaces and in manager–remote employee interaction. As recently suggested by Peirò et al. [64], “non-digital competencies will continue to be important, but many of them will have to be implemented in other ways in order to be effective, taking advantage of technological change and digitalisation” (p. 190). However, scholars’ interest in examining the requisite skills for supervisors in digital contexts is growing [9,10], and a definition of Digital Stress-Preventive Management Competencies is lacking in the literature, which will be introduced here.

### 1.2. Conceptualization of Digital, Virtual and E-Leadership and the Digital Stress-Preventive Management Competencies

Digital leadership is the umbrella term adopted in the literature, comprising terms such as e-leadership or virtual leadership, which have similar meanings and have been used interchangeably to define the supervisor’s ICT-mediated influence on employees [9,10]. Multidisciplinary scholars have extensively studied this multifaceted phenomenon, and the findings accumulated are heterogeneous and do not seem to converge within a clear picture [65,66]. The conceptual definition of e-leadership is one of the most adopted definitions by many authors (e.g., [67,68,69]), and it was the seminal paper of Avolio et al. in 2000 [70] that first conceptualized this construct. The authors defined e-leadership as “a social influence process mediated by information technology to produce a change in attitudes, feelings, thinking, behavior, and/or performance with individuals, groups, and/or organizations” ([70], p. 617). More recently, Van Wart et al. [16] defined e-leadership as the effective use and blending of electronic and traditional methods of communication. According to these authors, this construct implies an awareness of current ICT, the selective adoption of new ICT, and technical competence. Conversely, virtual leadership was conceptualized in line with the notion of virtual teams (“dispersed coworkers that are assembled using a combination of telecommunications and information technologies to accomplish an organizational task” [71], p. 17). Thus, the virtual leader is a “leader who is responsible for the management teams that are dispersed geographically and rely primarily upon electronic media for communication and collaboration” ([72], p. 15). Mindful of past definitions, our focus slightly differs from the above conceptualizations by targeting the work communication, design and organization of supervisors through ICT and employees’ well-being as a distal outcome. We define Digital Stress-Preventive Management Competencies as “the consolidated supervisors’ competencies of planning, organizing, setting objectives, creating and monitoring systems able to optimize a positive psychosocial work environment for remote workers, by organizing, communicating and managing work via ICT-mediated interactions”. See Table 1 for a glossary of definitions of digital, virtual and e-leadership.

### 1.3. The Present Study

Research on the manager’s role in well-being and health promotion is less developed. Models of “healthy leadership” are garnering increasing interest in occupational health science by contributing to a field of study often dominated by performance-driven models [41]. This domain is evident in the digital leadership literature, where existing research has predominantly focused on supervisors’ digital competencies to promote team performance (e.g., [11,12,13,14,15,16,17,18,19,20]). Additionally, the approaches explicitly focused on employees’ health promotion and psychosocial risk prevention as supervisors’ tasks do not yet consider the digitalization of work. Therefore, this study aims to develop and validate a tool to operationalize Digital Stress-Preventive Management Competencies, to be adopted for future practical (i.e., organizational intervention) and theoretical (i.e., research) applications.

To achieve the primary study aim, we designed three hierarchical study objectives. First, we will identify the management behaviors that can optimize the psychosocial work environment and well-being perceived by remote workers and develop a tool to measure the latent construct (i.e., Digital Management Competencies Indicator Tool—DMCIT). Second, we will validate the tool within organizational interventions context. Third, we will explore the concurrent validity of the new tool by investigating its relationship with the psychosocial work environment perceived by remote workers. This link will be tested first with only employees’ data and then by combining managers’ self-perceptions with the team’s perception of DMCs, thus following best practices on leadership research and employing multisource data and manager–team comparisons to maximize the findings’ exploratory power [50,51,52,53,54,55,56,57,58,59,60].

## 2. Method

This study follows a three-phase mixed-methods research design, integrating qualitative and quantitative approaches to ensure a comprehensive investigation of Digital Stress-Preventive Management Competencies (DMCs). The methodology was developed in accordance with best practices for scale development [73,74,75]. First, a preliminary exploratory phase consists of tool development and propaedeutically anticipates the second phase devoted to tool validation. Consequently, Phase 3 investigated the concurrent validity of the tool. Here follows a detailed description:

Phase 1, Tool Development: This consisted of three propaedeutic steps. First, we performed a literature review (1a) on digital competencies and in-depth semi-structured interviews with experts in hybrid or full remote team management via ICT (1b). The interviews were designed to elicit both the functional and dysfunctional ICT-mediated behaviors of managers in communicating, managing and organizing work. To achieve this goal, we used the critical incident technique by Flanagan [76]—the procedure used to collect direct observations of human behavior to facilitate their potential usefulness in solving practical problems and developing broad psychological principles. In doing so, we adopted both a deductive (i.e., literature review) and inductive (i.e., interview) approach [74]. The findings of both sources (literature and interviews) were first content-analyzed [77] in order to identify the critical themes and relevant behaviors and then adopted for the item generation process (2a), followed by an examination of the content validity of the items (2b) (which together composed our second step of Phase 1). The content validity was tested with six subjects (four lay experts and two subjects of the target population). As the last and third step, we conceptualized the overall findings of Phase 1 into two key Digital Stress-Preventive Management Competencies (3a), and then, grounding into the content of the competencies, a link with the psychosocial factors of the Management Standards approach [49,50] was supposed for the exploration of Phase 3 (3b). As a result, we developed a conceptual model of two critical competencies (i.e., supportive ICT-mediated interaction and avoidance of abusive ICT adoption) affecting four psychosocial factors of remote workers (i.e., superior support, role, demands and control).

Phase 2, Tool Validation: A two-step procedure was designed to test the soundness of the new measure psychometrically. Following guidelines on new measure validation [74], we first performed an exploratory factor analysis (EFA) and then a confirmatory factor analysis (CFA). EFA was performed to reduce the number of items and identify the tool’s potential underlying dimensions [78]. CFA was performed to review the overall psychometric properties of the new scale. The sample comprised remote workers involved in organizational interventions to develop their supervisors’ stress-preventive management competencies to promote organizational well-being. A total of 247 remote workers (RWs) adequately filled out an online survey of 15 min, and two independent randomized samples were created for EFA (103 RWs, 40% of the sample) and CFA (144 RWs, 60% of the sample). Particularly, the sample consisted of employees from three Italian public administrations, and the data was from time 1 data collection (i.e., pre-intervention).

**Figure 1:**
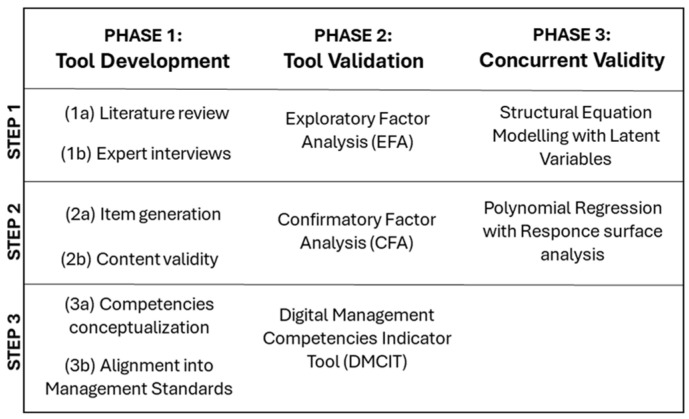
Graphical presentation of the method adopted.

Phase 3, Concurrent Validity: To conclude, we investigated the association between Digital Stress-Preventive Management Competencies (DMCs) and the psychosocial work environment of remote workers. To measure the psychosocial factors, we used the Stress Management Indicator Tool [79,80,81] of the Management Standards approach [49,50]. The concurrent validity was explored with two complementary analyses. First, from an employee’s point of view, we tested the association between DMCs and the psychosocial factors with structural equation modeling with latent variables on 247 RWs (Phase 2 sample). Then, to investigate the manager–team (in)congruence in DMCs and the relationship with psychosocial factors, we followed the recommendations for (in)congruence studies by performing several polynomial regressions with response surface analysis [82,83,84], as in other similar studies (e.g., [56,85]). This analysis enables us to examine the combined impact of two variables on a third while at the same time retaining information about the differences between the variables. The sample comprised 218 remote workers (part of the Phase 2 sample) plus their 50 managers. See Figure 1 for a graphical presentation of the method adopted.

## 3. Results

### 3.1. Phase 1: Tool Development

#### 3.1.1. Step 1a: Literature Review

The existing conceptualizations of key digital competencies are vague, suggesting that this field of study will continue to attract significantly more research as it has not yet entered its maturity stage [9]. However, targeting management competencies and organizing the competencies for similarity, six main key competencies are repeatedly outlined by scholars as relevant for the effective (digital) management of distributed teams:

(1) Digital communication: As for “traditional” (i.e., face-to-face) leadership, scholars widely agree that good communication is a crucial characteristic of leading digitally. This competence implies communicating via ICT effectively, managing communication flow in digital interactions, and the avoidance of over-communication [7,9,16,17,21,64,86,87,88,89,90]. In support of these assumptions, Wang et al. [91] reported that ineffective communication is a remarkable remote work challenge to tackle, and a survey of IT companies’ employees highlighted a positive relationship between the digital communication competence of supervisors reported by employees and their well-being [92].
(2) Technology knowledge and adoption: Considering digital transformation’s widespread evolution, some authors introduced the concept of “renaissance of technical skills” ([7] p. 13). The key characteristics commonly cited in the literature to define this competence are basic-technology savvy (mainly on ICT), appropriate ICT tool adoption and the ability to blend between traditional and virtual methods (such as face-to-face meetings and telephone and virtual conferencing) [7,10,16,17,18,88,89,93,94,95]. The latter was related to the well-being of IT companies’ employees concerning digital communication [92].
(3) Trust culture: Building trust rather than enacting controlling or commanding behaviors for remote workers’ management is a pivotal competence reported by many authors (e.g., [9,21,88,89,90,93]). Transparency, honesty, and general integrity have been outlined as prerequisites for building trust in distributed teams [9,16]. Moreover, the negative counterpart of the digital “building trust” competence of distributed managers (i.e., over-controlling/monitoring behaviors) was recently outlined as a techno-stress-increasing leadership characteristic [2].
(4) Support: Supervisors’ support for digital transformation [10,96], or generally ICT-mediated support [2,15,17,21,25,93], was reported by many scholars. Remarkably, supervisors’ (digital) support is a commonly cited pivotal well-being-oriented behavior for employees’. Karani and Mehta [97] showed that supervisor support of employees who worked from home during the COVID-19 pandemic was positively associated with employees’ well-being; Claassen et al. [25] explicitly focused on health-oriented leadership and developed a bottom-up digital leadership scale focused on support (some items: “My digital literacy is encouraged by my manager”; “I am supported by my manager to better understand and use digital applications”); Bentley et al. [98] highlighted that perceived support from leaders was negatively associated with the psychological strain of teleworkers; and Rademaker et al. [2] concluded their systemic review on leadership and techno-stress by inserting supportive leadership as a techno-stress-decreasing leadership characteristic.
(5) Foster collaboration: To strengthen the cooperation among distributed workers via ICT, the following factors are important: ensuring that teams use robust interaction-inclusive methods and fostering virtual team participation [16,94]; managing connectivity [7] by enabling and leading networks [99]; and special managerial attention to giving more feedback [21,88,89]. These were the best practices suggested for the effective digital management of remote workers. These assumptions are also supported by a qualitative interview study with distributed managers and employees by Poulsen and Ipsen [100], which revealed that continuous dialogue and feedback were beneficial for remote workers’ well-being.
(6) Respect for work–life boundaries: The pervasive proliferation of technology (namely digital ubiquity) has begun to be seriously discussed regarding workers’ well-being [2,101,102]. Considering this issue, several scholars highlighted supervisors’ pivotal attitude to respecting their team’s work–life boundaries [2,16,25,93]. Notably, special consideration was paid to not commissioning job demands outside working hours [2] and, more generally, to adopting the management attitude of not allowing virtual technologies to intrude into employees’ lives excessively [16]. This competence is linked to the empowerment/enslavement paradox related to the duality of digital working solutions and increasing flexibility and autonomy, which cause blurred boundaries between work and free time [103,104].

Overall, the literature review enabled a deductive conceptualization of key digital competencies and served as a foundation for analyzing the content of the interviews in the subsequent phase.

#### 3.1.2. Step 1b: Expert Interviews

**Sample and procedure**

We conducted 13 semi-structured interviews (average duration 1 h) with senior and line managers who were experts in digital work and remote employees’ management via ICT. Specifically, the participants were vital components of teams habitually practicing part-time remote work (i.e., typically 1–2 times per week), operating primarily in the human resources management field, and working in three Italian organizations (two public and one hybrid) with high maturity in terms of digital work and tools. The interviews were audio-recorded with the interviewees’ consent and transcribed for data analysis (see Supplementary Materials for questions).

Results:

Three main competencies were identified:

- ICT Tool Selection and Adoption: During the interviews, several managers outlined “digital laziness” as a critical risk for the digitalized work environment. This term was described as the inertia of supervisors (or collaborators) in over-adopting digital tools, avoiding face-to-face meetings or interactions, both in terms of co-presence days and also (perhaps more critically) when, even if present in the same location, online meetings are conducted without leaving the workstation. Moreover, experts suggested that choosing the right ICT tool or preferring face-to-face interaction instead (considering objectives and circumstances, as well as direct collaborators’ tech skills) is pivotal for employee management in digital workspaces. Thus, the first competence that emerged from the interview can be defined as “the skill to choose, or avoid adopting, the appropriate ICT tool to interact with remote workers considering objectives, circumstances and employees’ tech skills”. Here follows an explanatory sentence from interview n°11: “One of the problems with technology is this form of “digital laziness”, where technology is used simply because it exists, without considering its relevance. Instead, it should be employed for tasks that can be truly functional, and this decision must be carefully calibrated”.
- Remote team management: Among the best practices suggested by the experts, the interviewees encouraged the speed and promptness of feedback and the definition of digital conduct rules—for instance, by establishing a weekly update schedule for the whole team regardless of their members’ presence or remote work arrangement. Furthermore, interviewees highlighted that the manager’s availability for direct collaborators’ work emergencies enhances their significance even more in digital than face-to-face environments. Thus, “the skill to perform exhaustive communication, constant feedback and support for emergencies via ICT-mediated interaction” emerged as the second competence for virtual team management. In line with this, interviewee n°4 suggested the following best practices: “To provide timely information and ensure the involvement of everyone in updates. Do not focus only on those who are physically present while neglecting those working remotely, often forgotten”.
- Digital ubiquity management: Lastly, the experts agreed on the excellent resource digital connectivity represents for employees and work management. However, the pervasive proliferation of technology was also outlined as a substantial critical factor. Considering this, a fair level of awareness of risks and the potentiality of constant digital connectivity was outlined as a critical competence for managers in a well-being-oriented matter. Thus, respecting work–life boundaries and not commissioning job demands outside working hours, holidays or illness days was highlighted as a critical success factor. In line with this, a commonly cited critical situation was the supervisor’s over-monitoring behaviors (i.e., excessive random calls or control messages). Therefore, the third competence that emerged from the interviews can be defined as “the skill to respect team members’ work–life boundaries and the avoidance of over-monitoring behaviours”. Among functional behaviors, interviewee n°5 reported, “To avoid disturbing people at home. For example, if a team member is on vacation or sick, one might think, <<Come on, how hard is it to connect for a moment?>> This poses a significant risk. I am very mindful of this, but it is easy to slip into negative behaviours”.

Thus, adopting a practitioner-oriented and inductive perspective, these results highlighted which daily manager role-related behaviors carried out through ICT may be critical for remote workers’ well-being.

#### 3.1.3. Step 2: Item Generation and Content Analysis

- **Item Generation:** Following literature guidelines [74,75], seven principles were established to guide item generation: (1) Items must refer to observable behavior; (2) The item must be concise, specific, and unambiguous; (3) The item should include only active verbs, where the subject (the manager) acts; (4) To avoid the use of technical language; (5) To avoid the use of frequency adverbs, such as “frequently”, “often”, “rarely”, due to their subjective interpretability; (6) to make item sentences as short as possible; and (7) to develop significantly more items than necessary. As a result, a pool of 43 items was generated. Some items describe manager behaviors explicitly as ICT-mediated (i.e., Sends messages/emails with work-related requests outside of working hours). In contrast, others imply it (i.e., Provides necessary feedback to carry out work when working remotely).
- **Content Validity:** A sample of four lay experts (practitioners of human resources management with expertise in digitalization, remote work and training) and two subjects of the target populations (remote workers managed by a supervisor) filled out a content validity questionnaire. All subjects rated each item on clarity and relevancy on a 1–4 Likert scale, where one indicates that the item is not at all relevant or clear, and four indicates that the item is very relevant or very clear. Then, following Roebiento et al. [105] and Zamanzadeh et al. [106], the content validity indexes (CVIs) were calculated for each item, and items with CVI below 0.70 were removed, between 0.70 and 0.90 were revised, and above 0.90 remained. A refinement version of the measure consisted of 11 items.

#### 3.1.4. Step 3: Conceptual Model

- **Conceptualization of competencies:** By combining the Competencies for Effective Digital Managers outlined by the literature with the expert insights that emerged from the interviews and the resulting items from step 2, we synthesize the findings and conceptualized two different Digital Stress-Preventive Management competencies:
  1. *Supportive ICT-mediated interaction* (SIMI): The first competence identified refers to the supervisors’ ability to (1) communicate clearly and in a well-organized manner via in email, messages and video-call; (2) choose correctly whether to interact face to face or via ICT, alongside objectives and circumstances; (3) provide constant and prompt feedback and updates to teams when working remotely as when working in co-presence; (4) foster team ICT-mediated collaboration, such as boarding team members in online meetings or shared files; and (5) be available for emergency.
  2. *Avoidance of Abusive ICT Adoption* (AAIA): The second competence identified refers to the consolidated supervisors’ ability to adopt ICT appropriately by avoiding (1) sending emails or performing sudden calls with work demands outside working hours (i.e., holidays, late night, illness), when it is not necessary (no emergency); or (2) performing over-monitoring behaviors devoted to controlling remote workers’ actual work.
- **Alignment with Management Standards:** Consequently, the conceptual models of the study were developed by explicitly referencing the Management Standards [49,50], as in similar studies [43,46]. Therefore, the content of the competencies identified was conceptually mapped into the psychosocial factors of the aforementioned approach. First, supportive ICT-mediated interaction (SIMI) was related to the psychosocial factors of supervisor support (i.e., the encouragement, sponsorship and resources provided by the supervisor) and role (i.e., whether people understand their role and responsibilities within the organization). This is because clear and well-organized ICT communication, a feedback culture and general support by supervisors expressed through SIMI might optimize understanding roles and objectives, as well as the superior support perceptions of remote workers. Furthermore, the appropriate ICT adoption of supervisors (i.e., The avoidance of abusive ICT adoption) was associated with the perceptions of control (i.e., how much the worker can schedule or control his/her work) and demands (i.e., workload and work patterns) of remote workers. This is because being constantly over-monitored by supervisors or receiving job demands outside of working hours might impact remote workers’ job autonomy and workload perceptions.

**Figure 2:**
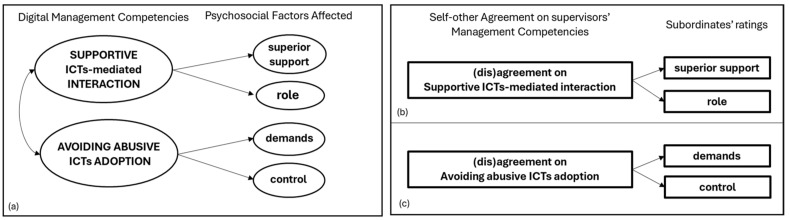
Conceptual models of the study. (**a**) Overall relationship between Digital stress-preventive management competencies and psychosocial factors, using employee data; (**b**) Individual relationship between manager-team (dis)agreement on SIMI and superior support and role; (**c**) Individual relationship between manager-team (dis)agreement on AAIA and demands and control.

Conversely, although the management of all psychosocial factors of the Management Standards approach is highly relevant for well-being [107,108], the content of peer support, change, and relationships does not seem to be significantly influenced by the competencies that emerged from our investigation. Therefore, the relationship between the two Digital Stress-Preventive Management Competencies (DMCs) and the related psychosocial factors affected by remote workers (i.e., supervisor support, role, demands and control) constitute the primary conceptual model of this study. This relationship will be investigated with employees’ data by Conceptual Model (a) and with manager–team (dis)agreement comparisons using Conceptual Models (b) and (c). Particularly, Conceptual Model (a) will investigate the overall relationship between supportive ICT-mediated interaction (SIMI) and supervisor support and role, as well as the contemporary relationship between the avoidance of abusive ICT adoption (AAIA) and demands and control. In comparison, Conceptual Models (b) and (b) will investigate the relationship between manager–team (dis)agreement’s impact on DMCs and psychosocial factors by singular analysis. The effect of manager–team (dis)agreement on SIMI will be explored using teams’ ratings of supervisor support and role [Conceptual Model (b)], while Conceptual Model (c) will explore the relationship between the impact of manager–team (dis)agreement on AAIA and the teams’ rating of demands and control (see Figure 2 for representations of the conceptual models).

### 3.2. Phase 2: Tool Development

Participants

The baseline sample consisted of 292 Italian public administration workers involved in organizational interventions. After excluding workers who did not engage in remote work, the Phase 2 sample comprised 247 employees (103 for EFA and 144 for CFA). On average, they worked 10.48 days per month (SD = 6.68) outside their primary workplaces, with 7.7% being fully remote. They collaborated in distributed teams with an average size of 8.43 members (SD = 4.95). We also inserted an attention check. This strategy allowed us to delete nine subjects from the preliminary sample. The socio-demographic information of the sample is unavailable as we decided, together with organizations, to protect participants’ anonymity in data collection. A five-point Likert scale was used: Strongly Disagree → Strongly Agree.

#### 3.2.1. Step 1: Exploratory Factor Analysis

*Procedure*

The EFA was performed using principal component analysis and varimax rotation with SPSS 23. The minimum factor loading criteria was set to 0.40. The commonality of the scale, which indicates the amount of variance in each dimension, was also assessed to ensure acceptable levels of exploration. Barlett’s Test of Sphericity was adopted to investigate the significance of the correlation matrix. It is recommended to have a range of 5 to 10 participants per item when conducting factor analysis [109]. However, the Kaiser–Meyer–Olkin measure was used to test sampling adequacy for factor analysis. In this regard, data with values above 0.80 are considered appropriate for factor analysis.

*Results*

Preliminary EFA analyses were run, and two items were removed [i.e., My manager in interactions mediated by digital communication tools (emails, messages, video calls, etc.) creates misunderstandings; and My manager communicates comprehensively by digital communication tools (emails, messages, etc.)]. The first was loaded into two factors, and the second was too similar to another item (i.e., D1); thus, the items with the best loading were chosen. The EFA results with the remaining nine items show that all commonalities were over 0.40. The result of Barlett’s Test of Sphericity was significant, *x*^2^ (*df* = 36) = 514.966 (*p* < 0.001), and the Kaiser–Meyer–Olkin measure was 0.82, which indicates suitability for factor analysis. The factor solution derived from the analysis yielded two factors, which accounted for 67.48% of the variation in the data. Factor 1’s initial eigenvalue was 4.7 (explaining data variance after rotation of 37.77%), and Factor 2’s initial eigenvalue was 1.30 (explaining data variance after rotation of 29,72%). The result confirmed the dimensional structure theoretically defined in the research. Factor 1 includes items from D1 to D5, and Factor 2 includes D6 to D9. Reliability indexes were adequate. Cronbach’s α was 0.88 for Factor 1 and 0.82 for Factor 2. See Table 2 for exploratory analysis results. Note that all items were developed in Italian and translated by a native Italian–English speaker to be reported in the present study. See Supplementary Materials for Italian items.

#### 3.2.2. Step 2: Confirmatory Factor Analysis

*Procedure*

A two-factor model was tested with CFA, with items D1–D5 loading on Factor 1 supportive ICT-mediated interaction, and with items D6–D9 loading on Factor 2, avoiding abusive ICT adoption. Model fit was evaluated using the following benchmarks: the root-mean-square error of approximation (RMSEA) and standardized root-mean-square residual (SRMR) should be less than 0.08, and the comparative fit index (CFI) and Tucker–Lewis index (TLI) should be greater than 0.90. All negative items of competence avoiding abusive ICT adoption were reversed prior to analysis to fit with the competence meaning and interpret the findings appropriately. The analyses were implemented using Mplus 8.

*Results*

The findings reveal good psychometric properties and model fit. The model tested fit adequately with the collected data (x^2^ = 643.43; df = 28; TLI = 0.94; CFI = 0.96; RMSEA = 0.08; SRMR = 0.04), the two predictors positively correlated (r = 0.57), and the reliability indexes were adequate. Cronbach’s α was 0.87 for Factor 1 and 0.83 for Factor 2 (see Table 3 for CFA results).

### 3.3. Phase 3: Concurrent Validity

#### 3.3.1. Structural Equation Modeling

*Participants and Procedure*

The sample of Phase 2 was used. Structural equation modeling with latent variables was used to test Conceptual Model (a). Single items were used as observed variables to build the latent variables of the two Digital Stress-Preventive Management Competencies (supportive ICT-mediated interaction and avoiding abusive ICT adoption, where negative items were reversed prior to analysis, as in Phase 2) and the four psychosocial factors of the study (i.e., superior support, role, demands and control). Superior support was measured with five items (e.g., I am given supportive feedback on the work I do), role was measured with three items (e.g., I am clear as to what my duties and responsibilities are), demands were measured with five negative items (e.g., I am pressured to work long hours) and control was measured with four items (e.g., I have a choice in deciding how I do my work) from the Italian adaptation of the Stress Management Indicator Tool [79,80,81,82,83,84,85,86,87,88,89,90,91]. The main analyses were implemented using Mplus 8. Preliminary descriptive and correlational analyses were conducted using SPSS 23.

*Results*

Descriptive statistics manifested significant and positive correlations among all study variables (note that negative items measuring demands and AAIA were reversed prior to descriptive and correlation analysis). The two DMCs highlight a correlation index of 0.52, and all psychosocial factors accounted for in this study correlate from 0.18 (demands-control) to 0.53 (demands-role). The strongest correlation (competence-psychosocial factors) was found between supervisor support and supportive ICT-mediated interaction (0.77). Reliability indexes were adequate for all study variables and are presented in Table 4 with means, SD and correlations’ coefficient.

**Figure 3:**
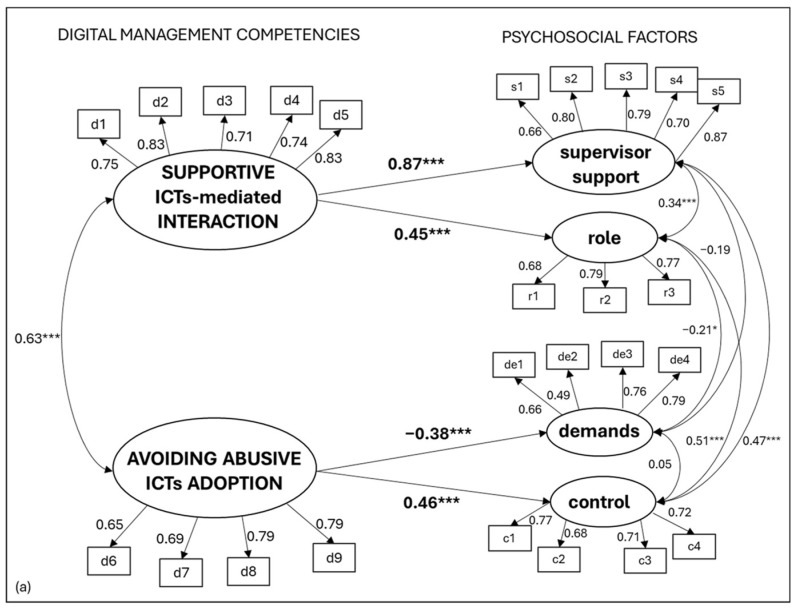
Conceptual Model (a) results. Note: *** = *p* < 0.001; * = *p* < 0.05.

The model tested fit adequately with the collected data (x^2^ = 493.62; df = 86; TLI = 0.91; CFI = 0.93; RMSEA = 0.06; SRMR = 0.06). The findings revealed that supportive ICT-mediated interaction was significantly and positively associated with role (β: 0.33, t = 5.55, *p* < 0.001) and superior support (β: 0.76, t = 9.16, *p* < 0.001). Moreover, the avoidance of abusive ICT adoption was significantly and positive related to control (β: 0.47, t = 7.28, *p* < 0.001) and negatively associated with perceptions of demands (β: −0.38, t = −5.40, *p* < 0.001). Thus, with increasing supportive ICT-mediated behaviors, the perception of role and supervisor supported increased, and with the increase in the avoidance of abusive ICT adoption, the perception of control increases and demands decreases in remote workers. See Figure 3 for a graphical representation of Conceptual Model (a) results.

#### 3.3.2. Manager–Team (Dis)agreement Investigation

*Participants and Procedure*

The baseline sample consisted of Phase 2 sample plus their 54 supervisors. Participants with missing data and teams composed of only one member were deleted to make manager–team inference. Thus, the final sample was composed of 218 remote workers and their 50 managers. Employees work on average 10.71 (SD = 6.54) days per month outside the main office location in a team composed of 9.76 members (SD = 6.67). The measures used were the same as in Section 3.3.1, with two differences: (1) managers filled the new tool in first person [e.g., D1 = I Communicate clearly through digital communication tools (email, messages, etc.)]; and (2) demands items were all reversed-scored to facilitate finding interpretation, such as high scores on demands means sustainable work demands, as opposed to Section 3.3.1.

To analyze the (in)congruence between predictors on outcomes, we used polynomial regression (PR) with response surface analysis (RSA). In this analysis, the two predictors (X and Y), their interactions (XY), and squared terms (X^2^ and Y^2^) are regressed on the outcome variable (Z) (i.e., PR). Then, the RSM uses PR estimates to 3D-plot the data distribution and to test the (in)congruence effect of the two predictors on the Z variable. This was carried out by calculating the five surface test values (a1, a2, a3, a4 and a5) based on unstandardized regression coefficients (i.e., b0, b1, b2, b3, b4, b5) and their significant difference from zero [82,83,84]. The first four surface test values represent the slopes and curvature of two lines. The first line (X = Y), the “line of congruence” (LOC), runs diagonally from the nearest to the farthest corners of the graph. a1 is the slope along the LOC and represents how the agreement between the predictors relates to the outcome. a2 is the curvature along the LOC and shows whether this relationship (between agreement and outcome) is linear or non-linear. The second line (X = −Y), called the “line of incongruence” (LOIC), runs diagonally from the left to the right corner. The slope along the LOIC is reflected by a3 and the curvature by a4. According to Shanock et al. [83], the first four surface values can be interpreted in isolation. Conversely, Nestler et al. [110,111] warn that three characteristics should be highlighted when concluding a congruence effect: (a) the LOC parameters should be nonsignificant; (b) the a4 must be negative and significant with a3 nonsignificant; and finally, (c) a5, which test the symmetry of the curvature, should be nonsignificant to conclude a strict congruence effect (the alignment between the congruence line and the first principal axis; see [110]). As recently suggested, we used both approaches to interpret our results for transparency [84].

Before starting the analysis, we examined whether the relevant statistics justified the aggregation of the employees’ ratings to the team level, as in similar studies (e.g., [85,112,113]), and we explored manager–team (in)congruence. As presented in Table 5, the analyses yielded a satisfactory ICC(1) and mean rWG(j) and thus supported the aggregation of the employees’ ratings. Moreover, because it is unavoidable to center predictors in this analysis [84,114], we scale-centered the predictors to use the same numerical number [115]. To conclude, four different PRs with RSA were performed. The first and second explored the relationship between the manager–team (dis)agreement on SIMI with superior support and role. The third and fourth focused on the manager–team (dis)agreement on AAIA with demands and control. Preliminary descriptive, correlational analyses and primary analyses were conducted using SPSS 23.

*Results*

The descriptive statistics and bivariate correlations are reported in Table 5. The correlations between managers’ and employees’ ratings of the two management competencies behaviors were all non-significant, indicating that variation exists between the ratings of self and others and that (in)congruence analyses were warranted. However, employees’ perceptions of digital competencies significantly positively correlate with each other and all the psychosocial factors (coefficients from 0.35–0.87). Reliability was optimal for employees ‘data and only adequate for managers’ self-reported competencies. This can be influenced by sample size (n = 50).

All four polynomial regressions explained significant variance (*R*^2^) in the outcomes; therefore, the examination of response surface analysis and surface tests a1 to a5 were guaranteed [83]. Particularly, the manager–team (in)congruence on SIMI significantly explained 77% and 53% of the variance for superior support and role, respectively. Meanwhile, the manager–team (in)congruence on AAIA significantly explained 24% and 33% of the variance for demands and control. Moreover, according to Nestler et al. [110], we cannot infer any strict congruence effect. See Table 6 for all PR and RSA results.

**Figure 4:**
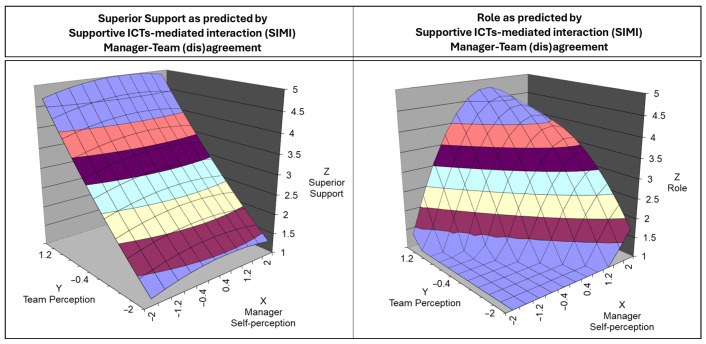
The 3-D graph of the surface response analysis for the manager–team (in)congruence on supportive ICT-mediated interaction in predicting superior support (**left**) and role (**right**).

Regarding manger–team (in)congruence on SIMI and role, the slope along the line of congruence (LOC) (a1 = 3.30, t = 4.36, *p* < 0.001) was positive and significant, suggesting that when managers and employees agree on high levels of SIMI, employees report greater clarity in their roles. The curvature along the LOC (a2 = −1.21, t = −3.64, *p* < 0.01) was significant and negative, indicating a dome-shaped effect where moderate agreement leads to the highest role clarity, while extreme agreement, either too high or too low, weakens this effect. For SIMI and superior support, the LOC slope (a1 = 0.93, t = 1.55, *p* = 0.12) was positive but not significant, suggesting that agreement on high SIMI does not systematically predict better employees’ perceptions of supervisor support than agreement on low SIMI. However, the response surface analysis (see Figure 4) showed that the relationship follows a flat plane, indicating that as both manager and team perceptions of SIMI increase, there appears to be a trend toward higher superior support, even if this effect is not statistically significant.

Examining the line of incongruence (LOIC) for SIMI, no significant effects were found for either role or superior support. The LOIC slope for superior support (a3 = −0.83, t = −1.47, *p* = 0.14) was negative but not significant, suggesting that whether managers overestimate or underestimate their own SIMI behaviors, it does not meaningfully change employees’ perceptions of supervisor support. Similarly, the LOIC slope for role (a3 = 0.12, t = 0.16, *p* = 0.87) was non-significant.

Regarding the manager–team (dis)agreement on avoidance of abusive ICT adoption (AAIA) in predicting demands, no significance was found regarding the slope and the curvature along the line of congruence (a1 = −0.06, t = −0.06, *p* = 0.95; a2 = 0.21, t = 0.56, *p* = 0.57). These results highlighted no significant difference between manager–team agreement level on AAIA predicting demands. Furthermore, the LOIC’s slope was insignificant (a3 = 0.57, t = 0.71, *p* = 0.47), suggesting no difference between high-low vs. low-high manager–team ratings on AAIA predicting demands.

**Figure 5:**
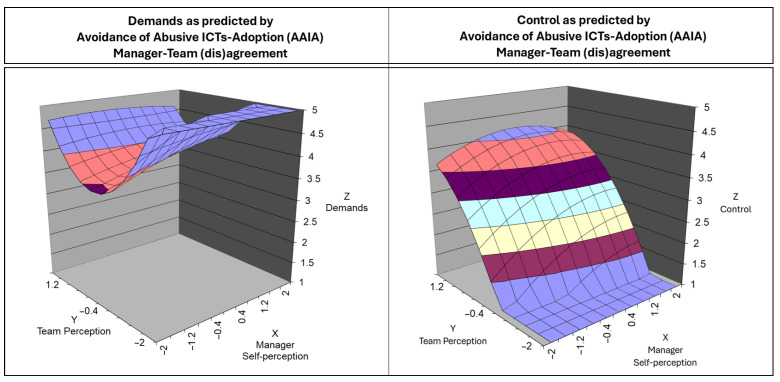
The 3D graph of the surface response analysis for the manager–team (in)congruence on avoiding abusive ICT adoption competence in predicting demands (**left**) and control (**right**).

For AAIA and job control, the LOC slope (a1 = 1.73, t = 2.12, *p* < 0.05) was positive and significant, indicating that when managers and employees agree on high levels of AAIA, employees perceive greater autonomy. Unlike SIMI and role clarity, the curvature along the LOC (a2 = −0.52, t = −1.61, *p* = 0.11) was not significant, meaning this relationship follows a linear trend rather than a dome-shaped one. However, similarly to SIMI, no significant effects were found along the LOIC for AAIA. The slope for control (a3 = −1.07, t = −1.53, *p* = 0.13) was negative but not significant, meaning that discrepancies between managers and employees do not substantially impact employees’ sense of job control. Figure 5 reports the 3D-surface plot of the manager–team (in)congruence on AAIA in predicting demands and control.

## 4. Discussion

To date, three approaches have been developed to capture the supervisor’s role in employees’ psychosocial risk and work-related stress prevention [21,45,48]. Even if they make a sound contribution to occupational health science, they do not cover the fast and recent digital transformation of work, in which the managers’ role is essential for employee well-being. To bridge this gap, the present study conducted a three-phase mixed method (e.g., interviews, content analysis, structural equation modeling) and multi-perspective investigation (e.g., experts, remote workers, and managers) to identify the digital stress-preventive management behaviors and develop a related indicator tool as a contribution to stress-preventive management competencies frameworks.

Phase 1 conceptualized the competencies and their link with psychosocial factors and consequently developed a tool to measure the latent constructs. As a result, two competencies emerged: supportive ICT-mediated interaction (SIMI) associated with superior support and role, and the avoidance of abusive ICT adoption (AAIA) related to the psychosocial perceptions of demands and control. Specifically, SIMI refers to the supervisors’ competence to communicate clearly and well-organized via ICT, correctly choosing whether to use ICT, providing constant and prompt feedback/updates to team members, and being available for emergencies. This latter is coherent with previous research that highlighted the remarkable role of effective digital communication [7,9,16,17,21,64,86,87,88,89,90], feedback culture [21,88,89], and supportive approach [2,16,17,21,25,93] for remote workers’ managers. Furthermore, one key SIMI behavior identified in this study overlaps with an item from MCPARS [46] (i.e., D3). This alignment is consistent with findings in the stress-prevention literature on management competencies (see [48]), reinforcing that these approaches examine the same underlying phenomenon. Meanwhile, the AAIA refers to the supervisors’ ability to adopt ICT appropriately by avoiding sending emails or performing sudden calls with work demands outside working hours (i.e., holidays, late night, illness), when it is not necessary (no emergency), or performing over-monitoring behaviors devoted to controlling remote workers’ actual work. Overall, the AAIA’s behaviors are strictly linked to the notion of digital ubiquity and align with previous research that reported the need for virtual teams’ managers to be able to build a trust culture [9,21,88,89,90,93], to avoid abusive and over-monitoring behaviors via ICT [2] and to respect work–life boundaries [2,16,25,93]. Notably, the competencies that emerged from Phase 1 of the study cover the main psychosocial factors that characterized remote work, such as work–life balance interference, extensive working hours/workload, constant availability expectations, autonomy and poor communication [116,117,118,119].

Subsequently, Phase 2 tested the developed tool with exploratory and confirmatory factor analysis on 247 RWs. The results supported a tool composed of nine items and two-factor solutions and provided a ready-to-use tool preparatory for exploring the concurrent validity. Therefore, Phase 3 comprised an employee-level analysis with structural equation modeling (SEM) and an (in)congruence investigation in which the self-ratings of the managers were compared with the employees‘ ratings in predicting and explaining the variance of outcomes of interest (i.e., superior support, role, demands, control).

The SEM confirmed the overall relationship between competencies and psychosocial factors. High team perceptions of digital management competencies were linked to a high level of superior support, role and control and low perceived job demands. This result was in line with previous studies that investigated the relationship between managers’ digital competencies and employees’ well-being outcomes [2,92,100]. Additionally, the manager–team (dis)agreement on SIMI and AAIA explained significant and important portion of variance in the outcomes and highlighted a more detailed relationship between competencies (multisource rated) and psychosocial factors rated by teams. In summary, manager–team agreement on SIMI is associated with higher role clarity, but the effect is dome-shaped, suggesting that extreme levels of agreement weaken its benefits. No significant effects were found for SIMI and superior support, although the response surface suggests a general increasing trend. Agreement on AAIA positively predicts job control in a linear fashion, while no effects were observed for job demands. Conversely, across all analyses, disagreement effects were not significant, indicating that misalignment between managers and employees does not systematically affect superior support, role clarity, job demands, or control. These results are coherent with manager–team (in)congruence investigation regarding “traditional” stress-preventive management competencies [56,57] but also performance-oriented framework and related outcomes (e.g., [113,120]). Notably, despite the lack of statistically significant results of disagreement, the response surface analysis graphs suggest different potential interpretation for the relationships between SIMI-superior support (Figure 4) and AAIA-control (Figure 5). Looking the surfaces, teams supervised by under-estimator managers reported more favorable psychosocial perceptions compared to those led by over-estimator managers. This pattern, visible in the response surface’s top-left (under-estimators) and bottom-right (over-estimators) quadrants, might indicate an underlying effect that was not detected due to sample limitations (e.g., under-representation of manager–team disagreement dyads). Additionally, quadratic polynomial regression assuming symmetry, which may not suit disagreement analysis (by definition asymmetric) and to overcome this issue the adoption of others statistical approaches able to capture the complex interplay between the disagreement on predictors and related outcomes were suggested (i.e., spline regression [121] or cubic polynomial regression [115]).

***Strengths and Limitations***

This study’s strengths include its mixed-method approach, incorporating qualitative and quantitative data analyses. The use of multi-source data and the comparison of different perceptions further enhanced the robustness of the findings and reduced the risk of common method bias [122], also improved by recommended statistical analysis (i.e., polynomial regression and response surface analysis) [82,83,84]. Additionally, data were collected from various organizational settings, specifically workers from three Italian public administrations, which were included in the sample within organizational interventions. This ensured that participants knew the data would be used for training, research, and consulting purposes as described in their informed consent, adding relevance and contextual depth. Moreover, the study’s focus on public administration adds further value, as five out of the six organizations involved were public entities, with one hybrid organization. Given the differences between public and private sector managers [123], these findings offer relevant insights into digital stress-preventive leadership within the specific constraints and dynamics of the public sector.

However, the study also has six main limitations. First, the cross-sectional nature of the data collection restricts causal inference between predictors and outcomes. Moreover, as an exploratory study on Digital Stress-Preventive Management Competencies, it is based on the limited existing literature, and this poses a challenge to compare with other research. Third, even if the statistics for the factor analysis confirmed the sample suitability, the number of participants was appropriate but not marvelous. This limitation is reinforced by the context of data collection (organizational interventions) and the small number of items for factor analyses (nine). Fourth, a limitation shared with studies using statistical analysis to illuminate (dis)agreement, as well as the impact thereof, is that they fail to capture the subjective experience of those involved [112]. Thus, although this study’s analysis allows for an objective calculation of manager–team (in)congruence, we do not know how managers and teams experience the (dis)agreement. Fifth, the developed tool may not comprehensively assess Digital Stress-Preventive Management Competencies, potentially overlooking key psychosocial factors like peer support, change, and relationships. However, as observed with traditional stress-preventive competencies [51,55,56], the identified behaviors may influence the overall psychosocial work environment. Lastly, while the study aimed to identify management competencies for digital workspaces, our sample—both interviewed experts and hybrid workers—was primarily from hybrid settings, making our findings more applicable to these contexts. However, excluding the first SIMI scale item on choosing between face-to-face and ICT-based interactions, the remaining eight items are still relevant for fully remote workers and their reliability is appropriate to measure SIMI (Cronbach’s α = 0.82).

***Theoretical Implications***

The present study contributes simultaneously to the emerging literature on digital leadership and to the stress-prevention literature by introducing the novel concept of Digital Stress-Preventive Management Competencies (DMCs). This construct may enhance the understanding of the managers’ role in digital workspaces for optimizing employees’ psychosocial work environment. Specifically, by integrating the Management Standards framework [49,50] with digital leadership competencies, this study extends existing research on psychosocial risk and work-related stress in remote work settings. Aligning with prior research on traditional stress-preventive management competencies but extend them by integrating ICT-specific leadership behaviors. The results also support the context-dependent nature of leadership, demonstrating how digitalization reshapes the way managerial behaviors influence employee experiences [65]. Thus, contextual variables (in our case ICT-mediated interaction) should be considered in leadership–follower dynamics and human resource management outcomes of interest.

***Future Directions for Research***

Future research should explore the application of these findings in intervention design, implementation, and evaluation, assessing how Digital Stress-Preventive Management Competencies can be effectively integrated into leadership development programs and organizational policies aimed at improving remote work conditions. Additionally, further studies could examine how these competencies interact with existing frameworks, such as the Management Competencies for Preventing and Reducing Stress (MCPARS) framework [21], to determine whether combining different approaches enhances their effectiveness in promoting employee well-being. Another important avenue for research involves testing the model in private sector settings, considering the differences in managerial practices between public and private organizations. Future studies could also adopt multilevel methodologies to explore how digital stress-preventive competencies function at different organizational levels or apply longitudinal designs to assess the long-term impact of these competencies on employee well-being and organizational outcomes. Lastly, given the strong association between supervisors’ behaviors in digital settings and techno-stress perceived by remote workers [2], future studies should investigate how these competencies influence employees’ experiences of techno-stress and whether the development of these competencies can reduce techno-stress within teams following targeted interventions.

***Practical Implications***

The findings of this study offer multiple practical applications. First, this study developed and tested a conceptual model of Digital Stress-Preventive Management Competencies that might be trained in organizational intervention to optimize remote workers’ psychosocial factors and well-being. Specifically, the competencies identified can be used as training content but also, as a recommendation, in combination with their validated tool. The tool can be used as a self-reflection exercise for managers or as upward feedback with team data to enhance managers’ self-awareness regarding their management style in digital settings and, consequently, equip them to develop their competencies by identifying potential areas for improvement. Moreover, the tool can be employed as a diagnostic tool (e.g., performance, organizational health or development needs assessment) for organizations to assess their managers’ competencies in digital work environments. Furthermore, this study aligns with emerging legislative discussions on digital work, such as the “right to disconnect” laws recently enacted in several countries such as Australia, Italy, France, Germany, Argentina, and Mexico [124]. By promoting a structured approach to ICT-mediated interactions, the findings highlight the importance of balancing digital connectivity with employees’ autonomy and well-being. Therefore, a more in-depth reflection on this right is warranted in both academic and non-academic contexts.

## 5. Conclusions

In conclusion, this study identifies and validates two key Digital Stress-Preventive Management Competencies (DMCs), highlighting their relevance in fostering a healthy psychosocial work environment for remote employees. The development of the DMCIT tool provides a practical resource for assessing and improving managers’ behaviors in digital contexts. Our findings underscore the need for well-being-oriented leadership models, emphasizing that effective digital leadership goes beyond performance enhancement and must also consider employee health and stress prevention. While this study provides important insights, future research should explore longitudinal effects of these competencies and their impact on organizational outcomes. Additionally, integrating intervention-based research could further refine our understanding of how training programs enhance digital stress-preventive competencies. As organizations continue adapting to hybrid and remote work models, equipping managers with the right digital leadership skills will be essential for ensuring both productivity and employee well-being.

## Figures and Tables

**Table 1:** Primary definition of digital, virtual and e-leadership and the authors’ definition.

Authors	Glossary	Definition
Avolio et al. (2000) [70]	E-leadership	“a social influence process mediated by information technology to produce a change in attitudes, feelings, thinking, behaviour, and/or performance with individuals, groups, and/or organizations” (p. 617)
Van Wart et al. (2019) [16]	E-leadership	“E-leadership is the effective use and blending of electronic and traditional methods of communication. It implies an awareness of current ICTs, selective adoption of new ICTs for oneself and the organization, and technical competence in using those ICTs selected.” (p. 83)
Berry et al. (2014) [72]	Virtual leadership	“The virtual leader is a leader who is responsible for the management of employees or work groups who are dispersed geographically and rely primarily upon electronic media for communication and collaboration” (p. 15)
Karakose et al. (2021) [10]	Digital leadership	“An umbrella term that comprises leadership styles such as technology leadership, virtual leadership, e-leadership, and leadership 4.0., all of which share a similar meaning and are used interchangeably throughout the literature” (p. 3)
Authors’ definition	Digital Stress-Preventive Management Competencies	“The consolidated supervisors’ competencies of planning, organizing, setting objectives, creating and monitoring systems able to optimize a positive psychosocial work environment for remote workers, by organizing, communicating and managing work via ICT-mediated interactions”

**Table 2:** Final factor structure of the domain items (EFA).

Factor	F1	F2	α
**Factor 1: Supportive ICT-mediated interaction (SIMI)**			0.88
D1. Communicates clearly through digital communication tools (email, messages, etc.)	0.75	−0.32	
D2. Chooses correctly whether to interact face-to-face with team members and when to use digital communication tools (email, phone, etc.) based on circumstances and objective	0.81	−0.26	
D3. Returns my calls/emails promptly	0.79	−0.13	
D4. Provides necessary feedback to carry out work when working remotely	0.81	−0.24	
D5. Supports team members in emergencies when working remotely	0.81	−0.25	
**Factor 2: Avoiding abusive ICT adoption (AAIA)**			0.82
D6. Displays over-monitoring behaviors when both are NOT in the same office or organizational workspace *	−0.19	0.80	
D7. Sends messages/emails with work-related requests outside of working hours *	−0.19	0.72	
D8. Exaggerates in monitoring whether you are working when you are not both in the same office location of the organization *	−0.19	0.77	
D9. Disturbs team members (via digital communication tools) during sickness, vacation, or outside of working hours when NOT necessary *	−0.38	0.78	
Eigenvalue	3.99	1.13	
Percentage of total variance after rotation	37.77	29.72	

Note: Every item starts with “My manager”; * = item reverse; α = Cronbach a.

**Table 3:** Confirmatory factor analysis of the DMCIT items.

Factor	Item	β	SE	t	α
**Supportive ICT-mediated interaction (SIMI)**	D1. Communicates clearly through digital communication tools (email, messages, etc.)	0.77	0.04	19.24	0.87
	D2. Chooses correctly whether to interact face-to-face with team members and when to use digital communication tools (email, phone, etc.) based on circumstances and objective	0.89	0.02	31.58	
	D3. Returns my calls/emails promptly	0.69	0.05	13.74	
	D4. Provides necessary feedback to carry out work when working remotely	0.63	0.05	11.22	
	D5. Supports team members in emergencies when working remotely	0.75	0.04	17.28	
**Avoiding abusive ICT adoption** **(AAIA)**	D6. Displays over-monitoring behaviors when both are NOT in the same office or organizational workspace *	0.612	0.06	10.12	0.83
	D7. Sends messages/emails with work-related requests outside of working hours *	0.74	0.04	15.87	
	D8. Exaggerates in monitoring whether you are working when you are not both in the same office location of the organization *	0.838	0.03	22.19	
	D9. Disturbs team members (via digital communication tools) during sickness, vacation, or outside of working hours when NOT necessary *	0.81	0.04	20.45	

Note: DMCIT = Digital Stress-Preventive Management Competencies Indicator Tool; β = standardized factor loadings; SE = standard error; t = t-value; * = item reverse; α = Cronbach a.

**Table 4:** Means, standard deviations (SD), Cronbach’s α and correlations among study variables for structural equation modeling investigation.

	Mean (SD)	1	2	3	4	5	α
**1. Supportive ICT-mediated interaction**	3.97 (0.67)	-					0.87
**2. Avoiding abusive ICT adoption**	4.25 (0.63)	0.52 ***	-				0.83
**3. Demands**	4.11 (0.63)	0.29 ***	0.32 ***	-			0.76
**4. Control**	3.86 (0.63)	0.38 ***	0.40 ***	0.18 *	-		0.82
**5. Supervisors Support**	3.95 (0.74)	0.77 ***	0.45 ***	0.34 **	0.49 ***	-	0.89
**6. Role**	4.22 (0.65)	0.43 ***	0.32 ***	0.53 ***	0.52 ***	0.52 ***	0.80

Note: *** = *p* < 0.001; ** = *p* < 0.01; * = *p* < 0.05; α = Cronbach’s α.

**Table 5:** Means, standard deviations (SD), Cronbach’s alpha, correlations among study variables for (dis)agreement investigation and intraclass correlation coefficient (ICC), within group agreement rWG(j) for aggregate variables.

	Mean (SD)	1	2	3	4	5	6	7	ICC (1)	Mean rWG (j)	α
**1. Supportive ICT-mediated interaction (manger)**	4.20 (0.37)	-							-	-	0.58
**2. Avoiding abusive ICT adoption (manager)**	4.38 (0.59)	0.17	-						-	-	0.71
**3. Supportive ICT-mediated interaction (team)**	4.00 (0.41)	0.10	−0.10	-					0.56 **	0.83	0.87
**4. Avoiding abusive ICT adoption (team)**	4.29 (0.36)	0.11	−0.07	0.49 **	-				0.55 **	0.88	0.83
**5. Superior Support (team)**	3.97 (0.45)	0.03	−0.07	0.87 **	0.48 **	-			0.62 **	0.84	0.89
**6. Role (team)**	4.18 (0.40)	0.06	0.00	0.63 **	0.48 **	0.62 **	-		0.58 **	0.87	0.81
**7. Demands (team)**	4.15 (0.37)	0.02	−0.07	0.35 *	0.45 **	0.29 *	0.31 *	-	0.41 **	0.91	0.74
**8. Control (Team)**	3.83 (0.35)	−0.15	−0.12	0.54 **	0.53 **	0.60 **	0.67 **	0.25	0.53 **	0.87	0.82

Note: ** = *p* < 0.001; * = *p* < 0.01; α = Cronbach’s α.

**Table 6:** Polynomial regression analysis and surface values results of the study.

Predictor	Self-Other Agreement on Supportive ICT-Mediated Interaction (SIMI)		Self-Other Agreement on Avoiding Abusive ICT Adoption (AAIA)	
**Outcome**	**Superior Support**	**Role**	**Demands**	**Control**
	**B**	**B**	**B**	**B**
**constant**	3.04 ***	2.17 ***	3.87 ***	2.54 ***
**X (b_1_)**	0.05	1.71 **	0.25	0.33
**Y (b_2_)**	0.88 **	1.59 ***	−0.31	1.39 *
**X^2^ (b_3_)**	−0.05	−0.48 *	−0.09	−0.09
**XY(b_4_)**	0.00	−0.52	−0.02	−0.12
**Y^2^ (b_5_)**	0.04	−0.19	0.33	−0.29
**F**	29.38 ***	10.01 ***	2.83 *	4.25 **
**R^2^**	0.77	0.53	0.24	0.33
**Surface test**				
**α1 = (b_1_ + b_2_)**	0.93	3.30 ***	−0.06	1.73 *
**α2 = (b_3_ + b_4_ + b_5_)**	−0.01	−1.21 **	0.21	−0.52
**α3 = (b_1_ − b_2_)**	−0.83	0.12	0.57	−1.07
**α4 = (b_3_ − b_4_ + b_5_)**	−0.01	−0.15	0.25	0.27
**α5 = (b_3_ − b_5_)**	−0.09	−0.27	−0.42	0.20

Notes: X = Supervisor self-rating; Y = Team rating; α1 = slope of agreement line; α2 = curve of agreement line; α3 = slope of disagreement line; α4 = curve of disagreement line; α5 = curvature symmetry; *** = *p* < 0.001; ** = *p* < 0.01; * = *p* < 0.05.

## Data Availability

The data are not publicly accessible due to privacy concerns.

## Supplementary Materials

The following supporting information can be downloaded at: https://www.mdpi.com/article/10.3390/ijerph22020267/s1, A. Semi-structured interviews questions; B. Digital Management Competencies Indicator Tool–Italian version.
