# Supplementary material for: Digital Stress-Preventive Management Competencies: Definition, Identification and Tool Development for Research and Practice
Source: Int J Environ Res Public Health. 2025 Feb 12;22(2):267. doi: 10.3390/ijerph22020267 (PMC11855444; doi:10.3390/ijerph22020267)
Supplement: Supplementary file 1 [file ijerph-22-00267-s001.zip › ijerph-3428889-supplementary.pdf]

SUPPLEMENTARY MATERIALS

Title: Digital Stress-preventive Management Competencies: definition, identification and tool development for research and practice

Authors: Glauco Cioffi, Cristian Balducci and Stefano Toderi

A. SEMI-STRUCTURED INTERVIEW QUESTIONS

(1) What are the enabling factors and resources that positively impact your team while working remotely or in ICTs-mediated interaction?

(2) What critical factors can adversely affect your team when working remotely or in ICTs-mediated interaction?

(3) Could you provide an example of a managerial situation where you effectively managed requests and/or work pressures from a team member or the entire team while working remotely or in ICTs-mediated interaction?

(4) Could you provide an instance where your response was less effective in addressing the pressures and demands of a team member or the entire team during remote work or in an ICTs-mediated interaction?

(5) Have you ever experienced a situation where you believe something you did or did not do had implications for your team, either in whole or in part, while working remotely or in ICTs-mediated interaction?

(6) Do you employ any other strategies or behaviours to enhance motivation or well-being within your team during remote work or ICTs-mediated interaction?

For questions 3, 4 and 5, when respondents actively answered, more specific information was asked: *What exactly did you do? Why did you decide to act in this way? How did the team member/team respond to this? How did you ascertain that your action was effective? Why do you believe this action was (in)effective in managing the situation?*

B. Digital Management Competencies Indicator Tool – Italian version

	**Supportive ICTs-mediated interaction**
D1	Comunica in maniera chiara tramite gli strumenti di comunicazione digitale (e-mail, messaggi ecc.)
D2	Sceglie correttamente quando interagire con i membri del team faccia a faccia e quando, invece, utilizzare strumenti di comunicazione digitale (e-mail, telefono ecc.) in base a circostanze e obiettivo
D3	Risponde prontamente alle mie telefonate/mail
D4.	Fornisce i feedback necessari per svolgere il suo lavoro quando lavorate a distanza
D5	Supporta i membri del team nelle situazioni di emergenza quando lavorate a distanza
	**Avoiding abusive ICTs-adoption**
D6.	Adotta comportamenti di controllo eccessivo quando NON siete entrambi nella stessa sede/ ufficio dell'organizzazione*
D7.	Manda messaggi/e-mail con richieste lavorative fuori orario lavorativo*
D8.	Esagera nel controllare se Lei lavora quando non siete entrambi nella stessa sede/ ufficio dell'organizzazione*
D9.	Disturba i membri del team (tramite strumenti di comunicazione digitale) in malattia, in ferie o fuori orario lavorativo quando NON necessario*
Note: *=item reverse	
